# Guidance for Using Tafenoquine for Prevention and Antirelapse Therapy for Malaria — United States, 2019

**DOI:** 10.15585/mmwr.mm6846a4

**Published:** 2019-11-22

**Authors:** Julia C. Haston, Jimee Hwang, Kathrine R. Tan

**Affiliations:** ^1^Department of Pediatrics, Emory University School of Medicine, Atlanta, Georgia; ^2^Division of Parasitic Diseases and Malaria, Center for Global Health, CDC; ^3^U.S. President’s Malaria Initiative, Washington, DC.

An estimated 219 million cases of malaria occurred worldwide in 2017, causing approximately 435,000 deaths (*1*). Malaria is caused by intraerythrocytic protozoa of the genus *Plasmodium* transmitted to humans through the bite of an infective *Anopheles* mosquito. Five *Plasmodium* species that regularly cause illness in humans are *P. falciparum*, *P. vivax, P. ovale*, *P. malariae,* and *P. knowlesi* (*2*). The parasite first develops in the liver before infecting red blood cells. Travelers to areas with endemic malaria can prevent malaria by taking chemoprophylaxis. However, most antimalarials do not kill the liver stages of the parasite, including hypnozoites that cause relapses of disease caused by *P. vivax* or *P. ovale*. Therefore, patients with these relapsing species must be treated with two medications: one for the acute infection, and another to treat the hypnozoites (antirelapse therapy). Until recently, primaquine was the only drug available worldwide to kill hypnozoites. Tafenoquine, a long-acting 8-aminoquinoline drug related to primaquine, was approved by the Food and Drug Administration (FDA) on July 20, 2018, for antirelapse therapy (Krintafel) and August 8, 2018, for chemoprophylaxis (Arakoda) (*3*,*4*). This report reviews evidence for the efficacy and safety of tafenoquine and provides CDC guidance for clinicians who prescribe chemoprophylaxis for travelers to areas with endemic malaria and treat malaria.

## Background

In 2016, a total of 2,078 imported malaria cases were reported in the United States; of the 1,853 (89.2%) cases with known species, 76.6% were caused by *P. falciparum*, 18.8% by *P. vivax* or *P. ovale,* and 4.5% by *P. malariae* or mixed infections (*5*). Plasmodium first develops in the liver before emerging up to 1 month later to infect red blood cells. Almost all antimalarials target only the blood stage of the parasite. Therefore, most chemoprophylaxis drugs are taken for 1 month after leaving the malaria area to allow the parasite to reach the targeted blood stage. However, *P. vivax* and *P. ovale* develop hypnozoites, a dormant stage of the parasite in the liver that can emerge months later to cause disease relapses. Treatment of these species requires antirelapse therapy (also known as radical treatment or radical cure). For travelers with intense or prolonged exposure to relapsing species of malaria, presumptive antirelapse therapy (PART) is recommended to kill hypnozoites (*6*). Until recently, only primaquine was used for this indication.

Tafenoquine, an 8-aminoquinoline drug related to primaquine, is only the second drug of its class to receive FDA approval. Tafenoquine kills both the liver and blood stages of the parasite, broadening its applicability for chemoprophylaxis to all species of malaria. FDA approved tafenoquine for prophylaxis of malaria in adults aged ≥18 years (Arakoda, 100 mg tablets) in August 2018 and antirelapse therapy of *P. vivax* malaria in persons aged ≥16 years (Krintafel, 150 mg tablets) in July 2018 (*7**,**8*). Like primaquine, tafenoquine can cause severe hemolysis in persons with glucose-6-phosphate dehydrogenase (G6PD) deficiency and quantitative G6PD testing is required before prescribing. Tafenoquine use is contraindicated in persons with G6PD deficiency (*9*).

This report summarizes the published efficacy and safety evidence for the recommended doses for both indications and provides guidance for the use of tafenoquine in the United States. A more comprehensive review of the literature on tafenoquine along with the biologic rationale for its use has been published elsewhere (*10*).

## Methods

CDC conducted a search of English-language articles available in PubMed, Ovid Medline, Scopus, and Global Health (CABDirect) on January 17, 2019, using keywords “tafenoquine or WR238605” and “prevent, prevention, prophylaxis, treatment, radical, or cure.” A total of 269 articles were collated and underwent title, abstract, and full text reviews by two physicians: an infectious disease specialist and a malaria subject matter expert. The references from review articles and meta-analyses, and the FDA labeling for both Krintafel and Arakoda were also reviewed to identify any additional studies. Randomized, double-blind, controlled trials performed in human subjects using the labeled recommended dosing regimens were preferentially included in the final review. Data on dosing regimen, outcome, and adverse events were abstracted. Based on the results of the review, CDC subject matter experts developed guidance for the use of tafenoquine.

## Rationale and Evidence

A total of 269 articles were identified. After excluding 232 during title review and 29 during abstract review, the eight remaining articles were reviewed fully and included in the analysis: five articles related to prophylaxis (*11*–*15*) and three to antirelapse (*16*–*18*); among these eight articles, seven included additional information for safety (*11*–*13*,*15*–*18*). All five studies cited by the FDA label were captured. An additional four peer-reviewed articles addressing the in vivo activity of hypnozoites of *P. ovale* and use of primaquine for *P. ovale* were reviewed (*6*,*19*–*21*); however, because they did not assess tafenoquine use, they were not included in the tafenoquine review.

**Prophylaxis.** Three of the five articles included were randomized controlled trials (RCTs), one was a reanalysis of data from an RCT, and one was a randomized human challenge study (*11*–*15*) (Table 1). Two RCTs compared tafenoquine (200 mg for 3 days, then weekly thereafter for up to 6 months) to placebo; both found a protective efficacy of 86% (95% confidence intervals [CIs] = 73–93 and 76–92) (*11*,*12*). Although not powered to detect statistical differences in efficacy, one of these RCTs described the efficacy between tafenoquine (protective efficacy = 86%, 95% CI = 76–92) and mefloquine (protective efficacy = 86%, 95% CI = 72–93) (*12*). The third RCT compared tafenoquine to mefloquine and observed no cases of malaria in either arm (*13*). The data from this study were reanalyzed in a separate study, using an estimation of attack rate, and found protective efficacy to be 100% (95% CI = 93–100) (*14*). These studies suggest comparable efficacy between tafenoquine and mefloquine, the current standard of care. Finally, the randomized human challenge study demonstrated 100% efficacy (95% CI = 40–100) of tafenoquine against the blood stage of *P. falciparum* in healthy volunteers compared with placebo (*15*).

**TABLE 1:** Findings from seven blinded, randomized trials of tafenoquine for prophylaxis and antirelapse treatment of malaria at recommended doses

Indication	Year published	Country (*Plasmodium* species)	Study population characteristics	Study length	Drug regimen	Sample size	Treatment	
							Outcome	% with outcome (95% CI)
Prophylaxis	2001*	Kenya (*P. falciparum* primarily)	Semi-immune	13 wks intervention, follow-up	TQ 200 mg x 3 days, then weekly	53	Protective efficacy	86 (73–93)
					Placebo	59		Reference
	2003^†^	Ghana (*P. falciparum* primarily)	Semi-immune	12 wks intervention, 4 wks additional follow-up (double-blind)	TQ 200 mg x 3 days, then weekly	91	Protective efficacy	86 (76–92)^§^
					MQ 250 mg/wk	46		86 (72–93)^§^
					Placebo	94		Reference
	2010^¶^	Timor-Leste (*P. falciparum* and *P. vivax*)	Nonimmune	6 mos intervention, follow-up 20 weeks	TQ 200 mg x 3 days, then weekly	492	No. of cases (protective efficacy)**	During intervention: 0 cases; During follow-up: 4 cases [100% (93–100)]^††^
					MQ 250 mg/wk	162		During intervention: 0; during follow-up: 1 case [100% (79–100)]
	2018^§§^	Australia (*P. falciparum* challenge)	Healthy, nonimmune	34 days	TQ 200 mg x 3 days, and 200 mg on day 10	12	Rescue treatment needed	0 (0–27)^¶¶^
					Placebo	4		100 (40–100)
Antirelapse therapy	2014***	Peru, India, Thailand, Brazil	≥16 yrs; microscopically confirmed *P. vivax* monoinfection	180 days from chloroquine initiation	CQ x 3 days + TQ 300 mg x 1	57	Relapse-free efficacy (ITT population)	89 (77–95)^†††^
					CQ x 3 days + PQ 15 mg x 14 days	50		77 (63–87)^†††^
					CQ x 3 days only	54		38 (23–52)
	2019^§§§^	Peru, Brazil, Colombia, Vietnam, Thailand	≥16 yrs; Hospitalized with microscopically confirmed *P. vivax* infection	180 days	CQ x 3 days + TQ 300 mg x 1	166	Recurrence-free efficacy (ITT population)	73 (65–79)
					CQ x 3 days + PQ 15 mg/day x 14 days	85		75 (64–83)
	2019^¶¶¶^	Peru, Brazil, Ethiopia, Cambodia, Thailand, Philippines	≥16 yrs (≥18 in Ethiopia); microscopically confirmed *P. vivax* infection	180 days	CQ x 3 days + TQ 300 mg x 1	260	Recurrence-free efficacy (ITT population)	62 (55–69)****
					CQ x 3 days + PQ 15 mg/day x 14 days	133		70 (60–77)
					Placebo	129		28 (20–36)

**Abbreviations:** CI = confidence interval; CQ = chloroquine; ITT = intention to treat; MQ = mefloquine; PQ = primaquine; TQ = tafenoquine.

* Shanks GD, Oloo AJ, Aleman GM, et al. A new primaquine analog, tafenoquine (WR 238605), for prophylaxis against *Plasmodium falciparum* malaria. Clin Infect Dis 2001;33:968–74.

^†^ Hale BR, Owusu-Agyei S, Fryauff DJ, et al. A randomized, double-blind, placebo-controlled, dose-ranging trial of tafenoquine for weekly prophylaxis against *Plasmodium falciparum*. Clin Infect Dis 2003;36:541–9.

^§^ Chi squared test (p<0.05).

^¶^ Nasveld PE, Edstein MD, Reid M, et al. Randomized, double-blind study of the safety, tolerability, and efficacy of tafenoquine versus mefloquine for malaria prophylaxis in nonimmune subjects. Antimicrob Agents Chemother 2010;54:792–8.

** Dow GS, McCarthy WF, Reid M, Smith B, Tang D, Shanks GD. A retrospective analysis of the protective efficacy of tafenoquine and mefloquine as prophylactic anti-malarials in non-immune individuals during deployment to an area with endemic malaria area. Malar J 2014;13:49.

^††^ Fisher exact TQ versus MQ p = 1.0.

^§§^ McCarthy JS, Smith B, Reid M, et al. Blood schizonticidal activity and safety of tafenoquine when administered as chemoprophylaxis to healthy, non-immune participants followed by blood stage *Plasmodium falciparum* challenge: a randomized, double-blinded, placebo-controlled Phase 1b study. Clin Infect Dis 2019;69:480–6.

^¶¶^ Fisher exact p<0.005.

*** Llanos-Cuentas A, Lacerda MV, Rueangweerayut R, et al. Tafenoquine plus chloroquine for the treatment and relapse prevention of *Plasmodium vivax* malaria (DETECTIVE Phase IIb): a multicentre, double-blind, randomized, phase 2b dose-selection study. Lancet 2014;383:1049–58.

^†††^ Log-rank TQ versus placebo p<0.0001; PQ versus placebo p = 0.0004.

^§§§^ Llanos-Cuentas A, Lacerda MVG, Hien TT, et al. Tafenoquine versus primaquine to prevent relapse of *Plasmodium vivax* malaria (GATHER). N Engl J Med 2019;380:229–41.

^¶¶¶^ Lacerda MVG, Llanos-Cuentas A, Krudsood S, et al. Single-dose tafenoquine to prevent relapse of *Plasmodium vivax* malaria (DETECTIVE Phase III). N Engl J Med 2019;380:215–28.

**** TQ hazard ratio (HR) 0.3; PQ HR 0.26, p<0.001.

**Antirelapse therapy.** One phase 2b randomized dose-selection trial and two phase 3 RCTs examined the efficacy of tafenoquine in the prevention of relapse in patients with confirmed *P. vivax* malaria at the labeled recommended regimen (Table 1) (*16*–*18*). Among these studies, tafenoquine was found to prevent relapse in 62%–89% of cases with a single 300 mg dose. In the large phase 2 dose-response study, efficacy of 300 mg and 600 mg were similar, and significantly higher than that of chloroquine alone (300 mg dose: 89.2%, 95% CI = 77–95, log-rank test p-value <0.001; 600 mg dose: 91.9%, 95% CI = 80–97, p<0.001; chloroquine: 36.5%, 95% CI = 23–52) (*16*).

***P. ovale* efficacy.** Tafenoquine is not labeled for use in *P. ovale*. Because *P. ovale* is relatively rare, accounting for fewer than 5% of malaria cases globally (*19*), it was not evaluated in the tafenoquine studies. Based on the biologic similarity of the hypnozoites of ovale and vivax, a CDC expert committee previously recommended the use of primaquine off-label for antirelapse therapy of *P. ovale* (*6*). With similar in vivo response of *P. ovale* to primaquine to that of *P. vivax* (*20*,*21*), CDC subject matter experts are extrapolating the use of tafenoquine to *P. ovale.*

**Safety.** Seven of the eight reviewed studies provided safety outcomes; four reported safety outcomes at the prophylaxis dose and three at the antirelapse therapy dose (Table 2) (*11*–*13*,*15*–*18*). Common adverse events included abdominal pain, constipation, diarrhea, vertigo, dizziness, sleep disturbances, and headache. Two studies described a nonsignificant increase in methemoglobin (*11*,*13*). Another reported asymptomatic decreases in hemoglobin, which resolved without intervention (*18*). One study described vortex keratopathy (a condition characterized by changes in the corneal epithelium resulting in a whorl pattern) in approximately 90% of patients receiving tafenoquine prophylaxis; the condition did not affect visual acuity and resolved within 1 year following drug discontinuation (*13*). Of note, persons with G6PD deficiency were excluded because 8-aminoquinolines can cause hemolytic anemia in these persons.

**TABLE 2:** Summary of key adverse events observed in persons receiving tafenoquine at recommended doses versus placebo or mefloquine

Year published	Study length	Drug regimen	Sample Size	Adverse event type reported, no. (%)					
				Gastrointestinal	Dermatologic	Neurologic	Ophthalmologic	Cardiac	Hematologic
**Prophylaxis dose**									
2001*	13 weeks intervention, follow up 4 wks	TQ 200 mg x 3 days, then weekly	55	Gastrointestinal 16 (29)	Any dermatologic 12 (22)	Neurologic 14 (26)	—	—	Methemoglobinemia, mean plateau concentrations 2.5%±1.6%
				-Abdominal pain 2 (4)	-Skin disorder 6 (11)	-Headache 13 (24)			
				-Constipation 4 (7)	-Rash 2 (4)				
				-Diarrhea 4 (7					
				-Gastritis 2(4)					
				-Gastroenteritis 3(6)					
		TQ 200 mg x 3 days	60	Gastrointestinal 20 (33)	Any dermatologic 12 (20)	Neurologic 11 (18)	—	—	—
				-Abdominal pain 1 (2)	-Skin disorder 5 (8)	-Headache 10 (17)			
				-Constipation 7 (12)	-Rash 1 (2)				
				-Diarrhea 4 (7)					
				-Gastritis 4 (7)					
				-Gastroenteritis 7 (12)					
		Placebo	61	Gastrointestinal 17 (28)	Any dermatologic 6 (8)	Neurologic 11 (18)	—	—	—
				-Abdominal pain 2 (3)	-Skin disorder 4 (7)	-Headache 11 (18)			
				-Constipation 3 (5)	-Rash 1 (2)				
				-Diarrhea 2 (3)					
				-Gastritis 4 (7)					
				-Gastroenteritis 5 (8)					
2003^†^	12 weeks intervention, 4 wks additional follow-up	TQ 200 mg x 3 days, then weekly	91	Elevated ALT 6 (6) ^§^	—	—	—	—	—
				Gastritis 5 (5)					
		MQ 250 mg/week	46	Elevated ALT 0	—	—	—	—	—
				Gastritis 1 (3)					
		Placebo	94	Elevated ALT 2 (2)	—	—	—	—	—
				Gastritis 2 (2)					
2010^¶^	6 mos intervention, follow-up 20 wks	TQ 200 mg x 3 days, then weekly	492	Severe gastrointestinal 8 (1) **	—	Neuropsychiatric 64 (13)^††^	Vortex keratopathy 69/74 (93) ^§§^	—	Methemoglobinemia, mean increase 1.8%
		MQ 250 mg/week	162	Severe gastrointestinal 0 (0)	—	Neuropsychiatric 23 (14)	Vortex keratopathy 0 (0)	—	Methemoglobinemia, mean increase 0.1%
2018 ^¶¶^	34 days after initiation of TQ (challenge study)	TQ 200mg x 3 days and then 200 mg on day 10	12	Abdominal discomfort 1 (8)	—	Headache 4 (33)	—	—	Hemoglobin decreased 2 (17)
				Abdominal pain 1 (8)		Hypoesthesia 0 (0)			
				Diarrhea 0 (0)		Lethargy 0 (0)			
				Dry mouth 1 (8)					
				Nausea 1 (8)					
				Vomiting 1 (8)					
		Placebo	4	Abdominal discomfort 1 (25)	—	Headache 4 (100)	—	—	Hemoglobin decreased 0 (0)
				Abdominal pain 0 (0)		Hypoesthesia 1 (25)			
				Diarrhea 1 (25)		Lethargy 1 (25)			
				Dry mouth 0 (0)					
				Nausea 3 (75)					
				Vomiting 2 (50)					
**Antirelapse therapy dose**									
2014***	Follow up to 180 days posttreatment	TQ 300 mg plus CQ	57	Upper abdominal pain 6 (11)	Pruritus 8 (14)	Asthenia 5 (9)	—	QT prolongation 3 (5)	Anemia 1 (2)
				Nausea 5 (9)		Insomnia 5 (9)			
		PQ 15 mg plus CQ	50	Upper abdominal pain 7 (14)	Pruritus 3 (6)	Asthenia 0 (0)	—	QT prolongation 5 (10)	Anemia 0 (0)
				Nausea 4 (8)		Insomnia 3 (6)			
		CQ only	54	Upper abdominal pain 5 (9)	Pruritus 7 (13)	Asthenia 0 (0)	—	QT prolongation 4 (7)	Anemia 0 (0)
				Nausea 3 (6)		Insomnia 1 (2)			
2019^†††^	Follow up to 180 days posttreatment	TQ 300 mg plus CQ	166	Nausea 16 (10)	Pruritus 20 (12)	Dizziness 27 (16)	Vortex keratopathy 1 (1)	—	—
				Vomiting 11 (7)		Headache 19 (11)	Retinal hypo-pigmentation 1 (1)		
							Retinal hyper-pigmentation 1 (1)		
		PQ 15 mg plus CQ	85	Nausea 6 (7)	Pruritus 19 (22)	Dizziness 13 (15)	Retinal hypo-pigmentation 1 (2)	—	—
				Vomiting 5 (6)		Headache 10 (12)			
2019 ^§§§^	Follow up to 180 days posttreatment	TQ 300 mg plus CQ	260	Nausea 16 (6)	Pruritus 127 (49)	Dizziness 22 (9)	Unilateral keratopathy 1	—	Hemoglobin decreased >3g/dL 14 (5)
				Vomiting 15 (6)		Headache 12 (5)	Unilateral retinal change 2		
				Diarrhea 10 (4)					
				Upper abdominal pain 8 (3)					
				Elevated ALT 6 (2)					
		PQ 15 mg plus CQ	129	Nausea 7 (5)	Pruritus 14 (11)	Dizziness 8 (6)	Retinal hypo-pigmentation 1	—	Hemoglobin decreased >3g/dL 2 (2)
				Vomiting 9 (7)		Headache 5 (4)			
				Diarrhea 2 (2)					
				Upper abdominal pain 6 (5)					
				Elevated ALT 3 (2)					
		CQ only	133	Nausea 9 (7)	Pruritus 17 (13)	Dizziness 4 (3)	—	—	Hemoglobin decreased >3g/dL 2 (2)
				Vomiting 7 (5)		Headache 9 (7)			
				Diarrhea 4 (3)					
				Upper abdominal pain 9 (7)					
				Elevated ALT 6 (5)					

**Abbreviations**: ALT = alanine aminotransferase; CQ = chloroquine; MQ = mefloquine; PQ = primaquine; TQ = tafenoquine.

* Shanks GD, Oloo AJ, Aleman GM, et al. A new primaquine analog, tafenoquine (WR 238605), for prophylaxis against *Plasmodium falciparum* malaria. Clin Infect Dis 2001;33:1968–74.

^†^ Hale BR, Owusu-Agyei S, Fryauff DJ, et al. A randomized, double-blind, placebo-controlled, dose-ranging trial of tafenoquine for weekly prophylaxis against *Plasmodium falciparum*. Clin Infect Dis 2003;36:541–9.

^§^ For all six, ALT exceeded a predetermined threshold and returned to normal levels when drug was discontinued. No clinical significance.

^¶^ Nasveld PE, Edstein MD, Reid M, et al. Randomized, double-blind study of the safety, tolerability, and efficacy of tafenoquine versus mefloquine for malaria prophylaxis in nonimmune subjects. Antimicrob Agents Chemother 2010;54:792–8.

** Most common gastrointestinal events: abdominal pain, constipation, and diarrhea. No difference between tafenoquine and mefloquine gastrointestinal events.

^††^ No difference between tafenoquine and mefloquine, and no severe neuropsychiatric events observed. Most common events were vertigo, dizziness, and sleep disorders. One tafenoquine subject withdrew because of depression (moderate), and one for hyperesthesia (moderate).

^§§^ Subset analysis for vortex keratopathy. Not associated with visual disturbances and resolved by 1 year.

^¶¶^ McCarthy JS, Smith B, Reid M, et al. Blood schizonticidal activity and safety of tafenoquine when administered as chemoprophylaxis to healthy, non-immune participants followed by blood stage *Plasmodium falciparum* challenge: a randomized, double-blinded, placebo-controlled Phase 1b study. Clin Infect Dis 2019;69:480–6.

*** Llanos-Cuentas A, Lacerda MV, Rueangweerayut R, Krudsood S, Gupta SK, Kochar SK, et al. Tafenoquine plus chloroquine for the treatment and relapse prevention of *Plasmodium vivax* malaria (DETECTIVE): a multicentre, double-blind, randomized, phase 2b dose-selection study. Lancet. 2014;383:1049–58.

^†††^ Llanos-Cuentas A, Lacerda MVG, Hien TT, et al. Tafenoquine versus primaquine to prevent relapse of *Plasmodium vivax* malaria (GATHER). N Engl J Med 2019;380:229–41.

^§§§^ Lacerda MVG, Llanos-Cuentas A, Krudsood S, et al. Single-dose tafenoquine to prevent relapse of *Plasmodium vivax* malaria (DETECTIVE Phase III). N Engl J Med 2019;380:215–28.

## Recommendations

Tafenoquine is an additional FDA-approved antimalarial option for malaria prophylaxis in adults aged ≥18 years, and for antirelapse therapy in persons aged ≥16 years (Box).

BOX Guidance for the use of tafenoquine by indication*

**Prophylaxis^†^**

- **Loading regimen**
  - 200 mg daily by mouth x 3 days before departure
- **Maintenance regimen**
  - 200 mg once weekly by mouth beginning 7 days after last loading dose
  - Continue for entire duration of travel plus one additional dose after returning
  - Take on the same day of the week each week

**Antirelapse therapy^§^**

- Single 300 mg dose by mouth, ideally on the first or second day of blood-stage treatment
- If not feasible to overlap with blood-stage treatment, may be taken as soon as possible afterwards

**Presumptive antirelapse therapy^§^**

- Single 300 mg dose by mouth, ideally on the same day as the last dose of prophylaxis
- If not feasible to overlap with last dose of prophylaxis, may be taken as soon as possible afterwards
- Antirelapse dose not needed if primaquine or tafenoquine is used for prophylaxis

* Contraindications: glucose-6-phosphate dehydrogenase (G6PD) deficiency, pregnancy, breastfeeding (if infant has G6PD deficiency or if G6PD status is unknown), known hypersensitivity to 8-aminoquinolines, history of psychiatric disorder.

^†^ Persons aged ≥18 years.

^§^ Persons aged ≥16 years.

**Dosage and indication.** In adults traveling to areas with malaria, tafenoquine (Arakoda, 100 mg tablets) can be used for chemoprophylaxis for all species of malaria. The prophylactic dose is 200 mg daily for the 3 days preceding the trip, 200 mg weekly during the trip, and a single 200 mg dose during the week after returning. In persons aged ≥16 years, tafenoquine (Krintafel, 150 mg tablets) can be used for presumptive antirelapse therapy or PART for *P. vivax* and off-label for *P. ovale*. The single 300 mg antirelapse or PART dose should ideally overlap with blood-stage treatment or the last dose of prophylaxis. If this is not feasible, tafenoquine may be taken as soon as possible afterwards. PART is not necessary if primaquine or tafenoquine is taken for primary prophylaxis. Tafenoquine should be administered with food.

**Contraindications and warnings.** Like primaquine, tafenoquine is contraindicated in persons with G6PD deficiency because it might cause hemolytic anemia. If G6PD status is unknown, quantitative G6PD testing must be performed to confirm normal activity before administration of tafenoquine. Qualitative G6PD testing might miss persons with intermediate deficiency and is inadequate to guide tafenoquine administration. Tafenoquine is contraindicated in pregnancy because of the unknown G6PD status of the fetus and should not be used in breastfeeding women if the infant has G6PD deficiency or if the infant’s G6PD status is unknown. Because psychiatric adverse reactions were observed in persons with a previous history of psychiatric conditions, tafenoquine should not be used in these persons (*3*,*4*), and other antimalarials could be considered for prophylaxis, or primaquine can be considered as an alternative for antirelapse therapy. Tafenoquine is contraindicated in persons with known hypersensitivity to 8-aminoquinolines.

**Adverse events and reporting.** Adverse events might be delayed in onset or duration because of tafenoquine’s long half-life. Common adverse reactions include dizziness, nausea, vomiting, and headache. When used for prophylaxis, elevated liver enzyme levels, insomnia, depression, abnormal dreams, and anxiety were also observed. Suspected adverse reactions can be reported to FDA via MedWatch at https://www.fda.gov/safety/medwatch.

## Discussion

This guidance regarding use of tafenoquine for both prophylaxis of all species of malaria and antirelapse therapy for *P. vivax* is consistent with FDA labeling. Recommendations for PART and antirelapse therapy of *P. ovale* are off-label. It is not feasible to conduct adequately powered clinical trials for *P. ovale* malaria because of its relatively low incidence. Therefore, evidence for efficacy against *P. vivax* was extrapolated to *P. ovale*.

For persons with contraindications to tafenoquine, other antimalarial options for malaria chemoprophylaxis and radical cure can be considered. There are several other options for chemoprophylaxis, each with its own contraindications and warnings, which can be used depending on the patient and drug-resistance in the areas of travel. These include atovaquone-proguanil, chloroquine, doxycycline, and mefloquine. For antirelapse therapy, the only alternative is primaquine. For nonpregnant persons with borderline or intermediate G6PD deficiency requiring antirelapse treatment, an alternative dosing regimen of primaquine could be considered at 45 mg (base) once weekly for 8 weeks, with close monitoring and consultation with an infectious disease expert. Persons with severe G6PD deficiency will require antimalarials at prophylaxis doses for 1 year instead of an 8-aminoquinoline (i.e., primaquine or tafenoquine). Pregnant women with normal G6PD levels, requiring antirelapse therapy could be given chloroquine at chemoprophylaxis doses (500 mg salt once weekly) until after delivery, and then an 8-aminoquinoline, depending on whether the woman is breastfeeding and the G6PD status of the infant.

The approval of tafenoquine marks a notable advancement for the prevention of malaria and treatment of *P. vivax* and *P. ovale*. Its long half-life of 15 days allows for weekly prophylactic dosing during travel and a single dose for antirelapse therapy, which has the potential to increase adherence for both indications (*7*,*8*). With two strengths of tafenoquine tablets available, it is important that clinicians ensure that the appropriate dose is used for each specific indication.

Malaria is a notifiable disease in the United States. CDC’s National Malaria Surveillance System collects information about cases of malaria occurring in the United States, providing an opportunity to assess the use and clinical outcomes of tafenoquine. Postmarketing surveillance is being conducted to monitor the occurrence of adverse events. Adverse events related to tafenoquine should be reported voluntarily to FDA’s MedWatch adverse event reporting system, and as part of routine reporting to CDC.

**Summary**

**What is already known about this topic?**

Malaria can be prevented by taking antimalarials when traveling to an area with malaria. Treatment of malaria caused by *Plasmodium vivax* and *Plasmodium ovale* requires antirelapse therapy to kill the dormant liver-stage parasite.

**What is added by this report?**

Adults aged ≥18 years can take tafenoquine (Arakoda 100 mg tablets) to prevent malaria. Persons aged ≥16 years requiring antirelapse therapy for *P. vivax* or *P. ovale* can take tafenoquine (Krintafel 150 mg tablets). Before using tafenoquine, quantitative testing to rule out glucose-6-phosphate dehydrogenase deficiency is required.

**What are the implications for public health practice?**

Tafenoquine is another option for malaria chemoprophylaxis and for antirelapse therapy. The simplified dosing regimen has the potential to improve adherence.
